# Supplementary material for: Efficacy of Wex-cide 128 disinfectant against multiple prion strains
Source: PLoS One. 2023 Aug 24;18(8):e0290325. doi: 10.1371/journal.pone.0290325 (PMC10449212; doi:10.1371/journal.pone.0290325)
Supplement: S1 Table — (DOCX) [file pone.0290325.s001.docx]

| Prion strain | Disinfectant | BH or wire expt. | Brain homogenate dilution group^a^ | | | | | | | |
| --- | --- | --- | --- | --- | --- | --- | --- | --- | --- | --- |
|  |  |  | 10^-1^ | 10^-3^ | 10^-4^ | 10^-5^ | 10^-6^ | 10^-7^ | 10^-8^ | 10^-9^ |
| 263K^b^ | saline | BH |  | ns | ns | ns | ns | ns | 1/4 | 1/4 |
|  | 40% Wex-cide | BH |  |  | 0/5 | ns | ns |  |  |  |
|  | 4% Wex-cide | BH |  | 0/6 | ns | ns | ns |  |  |  |
|  | 2% LpH | BH |  | 1/4 | 0/4 | ns |  |  |  |  |
|  | 4% Wex-cide aged 6 weeks | BH |  | 1/7^e^ | ns | ns |  |  |  |  |
|  | 2% LpH aged 6 weeks | BH |  | 1/8^e^ | ns | ns |  |  |  |  |
|  | 4% Wex-cide aged 8 months | BH |  | 1/8^e^ | ns | ns |  |  |  |  |
|  | 2% LpH aged 8 months | BH |  | 0/8 | ns | ns |  |  |  |  |
|  | 4% Wex-cide 2 min | wire | 0/4 |  |  |  |  |  |  |  |
|  | 4% Wex-cide 30 min | wire | 0/4 |  |  |  |  |  |  |  |
|  | 2% LpH 2 min | wire | 0/3 |  |  |  |  |  |  |  |
|  | 2% LpH 30 min | wire | 0/4 |  |  |  |  |  |  |  |
|  | none | wire | 1/1 |  | ns | ns | 3/3 | 1/8 |  |  |
| CWD^c^ | 4% Wex-cide | BH |  | 0/5 | 0/4 | ns |  |  |  |  |
|  | 2% LpH | BH |  | 0/5 | 0/4 | ns |  |  |  |  |
|  | water | BH |  | 4/4 |  | 1/1 | 2/2 | 3/4 |  |  |
| 22L^b^ | 4% Wex-cide | BH |  | 1/8 | ns |  |  |  |  |  |
|  | 2% LpH | BH |  | 3/3 | 1/8 |  |  |  |  |  |
|  | saline | BH |  | ns | ns | ns | ns | 2/2 | 1/4 | 0/4 |
| sCJD^d^ | saline | BH |  | ns | 4/4 | 3/4 | 0/4 | 0/4 |  |  |
|  | 40% Wex-cide | BH |  |  | 1/5 | 0/3 | ns |  |  |  |
|  | 4% Wex-cide | BH |  | 4/4 | 2/4 | 0/3 | 0/3 |  |  |  |
|  | 2% LpH | BH |  | 3/4 | 3/4 | 0/3 |  |  |  |  |
|  | 4% Wex-cide 2 min | wire | 1/6 |  |  |  |  |  |  |  |
|  | 4% Wex-cide 30 min | wire | 0/6 |  |  |  |  |  |  |  |
|  | 2% LpH 2 min | wire | 5/6 |  |  |  |  |  |  |  |
|  | 2% LpH 30 min | wire | 0/6 |  |  |  |  |  |  |  |
|  | none | wire | 3/3 | 2/3 | 1/1 | 4/4 |  |  |  |  |

**Table S1.** Summary of prion diagnostic assay results on brain tissues from bioassay mice.

^a^ The fraction reported indicates the number of mice scored positive (by prion screening test) over the number of mice tested. In some cases, not all the mice were tested from each group and total group sizes may not match the bioassay table numbers for this reason.

^b^ Brains were screened by immunoblot to confirm prion disease status.

^c^ Brains were screened by the RT-QuIC assay to confirm prion disease status.

^d^ Brains were screened by either IHC for prion deposition or the RT-QuIC assay to confirm prion disease status.

^e^ The positive mouse in this group was identified by immunoblot, but did not have clinical signs of prion disease at the termination of the experiment. For calculations of titers this mouse was scored as negative in bioassay table 2.

ns: not screened, indicates that no mice from this dilution were screened
